# Sociability in a non-captive macaque population is associated with beneficial gut bacteria

**DOI:** 10.3389/fmicb.2022.1032495

**Published:** 2022-11-11

**Authors:** Katerina V.-A. Johnson, Karli K. Watson, Robin I. M. Dunbar, Philip W. J. Burnet

**Affiliations:** ^1^Department of Psychiatry, University of Oxford, Oxford, United Kingdom; ^2^Department of Experimental Psychology, University of Oxford, Oxford, United Kingdom; ^3^Institute of Cognitive Science, University of Colorado Boulder, Boulder, CO, United States

**Keywords:** gut microbiome, microbiome–gut–brain axis, social behaviour, macaques, *Macaca mulatta*, grooming, immune status

## Abstract

The relationship between social behaviour and the microbiome is known to be reciprocal. Research in wild animal populations, particularly in primate social groups, has revealed the role that social interactions play in microbial transmission, whilst studies in laboratory animals have demonstrated that the gut microbiome can affect multiple aspects of behaviour, including social behaviour. Here we explore behavioural variation in a non-captive animal population with respect to the abundance of specific bacterial genera. Social behaviour based on grooming interactions is assessed in a population of rhesus macaques (*Macaca mulatta*), and combined with gut microbiome data. We focus our analyses on microbiome genera previously linked to sociability and autistic behaviours in rodents and humans. We show in this macaque population that some of these genera are also related to an individual’s propensity to engage in social interactions. Interestingly, we find that several of the genera positively related to sociability, such as *Faecalibacterium*, are well known for their beneficial effects on health and their anti-inflammatory properties. In contrast, the genus *Streptococcus*, which includes pathogenic species, is more abundant in less sociable macaques. Our results indicate that microorganisms whose abundance varies with individual social behaviour also have functional links to host immune status. Overall, these findings highlight the connections between social behaviour, microbiome composition, and health in an animal population.

## Introduction

The emergence of social systems is considered one of the major evolutionary transitions (Maynard Smith and Szathmáry, 1997). Social organisation is a fundamental characteristic of many animal populations (Strandburg-Peshkin et al., 2015; Johnson and Dunbar, 2016; Johnson et al., 2017; Wild et al., 2021). In particular, most primate species, including humans, live in complex social groups. Whilst group living comes with numerous benefits, such as the acquisition of resources, access to mates, and protection from predation (Silk, 2007), social networks can also facilitate the spread of disease (Krause and Ruxton, 2002). Until recently, most research has focused on the transmission of parasites and pathogens within animal social groups (Altizer et al., 2003; Craft, 2015), which may occur either directly, *via* interactions between individuals, or indirectly through their shared environment. However, the advent of microbiome research has revealed that the majority of host-associated symbionts are harmless and many may even be beneficial to health and survival. This has prompted studies exploring how the social transmission of microorganisms may influence microbiome composition, and the possible consequences for host fitness (Archie and Tung, 2015; Raulo et al., 2021; Worsley et al., 2021). Indeed, the mammalian gut microbiome can modulate host development, digestion, physiology, metabolism, immunity, and behaviour. The relationship between the microbiome and behaviour is therefore reciprocal, since social interactions can influence the gut microbiome, which in turn can affect social behaviour.

Recent research has shown the role that the host’s social environment can play in shaping an individual’s microbiome (Sarkar et al., 2020b). For example, in a wild baboon population (*Papio cynocephalus*), social group membership was found to be the strongest predictor of gut microbiome composition, with individuals that interacted more frequently through grooming having more similar gut microbial communities (Tung et al., 2015). Studies on various other primates, notably chimpanzees, lemurs, and howler monkeys, have also shown that different social groups within a species have distinct gut microbial communities (Degnan et al., 2012; Bennett et al., 2016; Amato et al., 2017; Perofsky et al., 2017; Rudolph et al., 2022) and that social interactions, such as grooming, promote microbial similarity between individuals (Moeller et al., 2016; Raulo et al., 2017). In addition, increased social contact has been associated with a more diverse gut microbiome in a range of animal populations (Li et al., 2016; Moeller et al., 2016; Billiet et al., 2017; Perofsky et al., 2017). Further evidence of socially mediated microbial transmission comes from a study of wild baboons (*P. cynocephalus*) showing that the gut microbiome of immigrant males in a social group changes over time to resemble the microbiome composition of long-term residents of the group (Grieneisen et al., 2017). There is also evidence that microorganisms are socially transmitted between humans since people living in the same household have more similar gut microbial communities (Yatsunenko et al., 2012; Song et al., 2013; Lax et al., 2014; Schloss et al., 2014; Odamaki et al., 2018) and individuals with a larger social network have a more diverse gut microbiome (Johnson, 2020). The role of social behaviour in influencing microbiome composition may therefore have implications for host health and fitness, since transmission of beneficial microorganisms and a more diverse microbiome may help to protect hosts from pathogens (Dillon et al., 2005; Koch and Schmid-Hempel, 2011; Lawley et al., 2012; Abt and Pamer, 2014) and improve the stability and resilience of the gut microbiome (Johnson and Burnet, 2016).

The reciprocal interaction between host behaviour and infection status is well known in parasitology (Ezenwa et al., 2016), whereby behaviour can influence parasite transmission (Altizer et al., 2003) and parasites can in turn alter the behaviour of their host (Moore, 2013; Johnson and Foster, 2018). Similarly, in addition to the role of social interactions in microbial transmission, recent research has demonstrated that the mammalian gut microbiome can affect various aspects of host behaviour, including anxiety, depressive-like behaviour, and sociability (Sharon et al., 2016; Johnson and Foster, 2018). In particular, studies manipulating the gut microbiome in rodents (using germ-free conditions, antibiotic treatment, or probiotic supplementation) have revealed that the gut microbiome influences neurochemistry, social development, and behaviour (Hsiao et al., 2013; Crumeyrolle-Arias et al., 2014; de Theije et al., 2014; Desbonnet et al., 2014, 2015; Buffington et al., 2016; Leclercq et al., 2017; Guida et al., 2018; Stilling et al., 2018; Johnson and Burnet, 2020; Sarkar et al., 2020a). These findings have motivated research into the gut microbiome in autism since its key feature is a deficit in social interactions (Krajmalnik-Brown et al., 2015) and moreover autism is frequently comorbid with gastrointestinal issues (Hsiao, 2014). Differences in gut microbiome composition between autistic and neurotypical controls have been found repeatedly in human studies, indicating that autism is associated with an altered gut microbial community (Vuong and Hsiao, 2017). These differences likely represent a two-way relationship whereby their environment, limited social interactions and lifestyle impact the gut microbiome and their microbiome also contributes to their behavioural traits. In fact, a recent study involving faecal microbiota transplantation in children with autism showed that it improved multiple aspects of behaviour, including social behaviour, as well as ameliorating gastrointestinal symptoms (Kang et al., 2017).

Here, we investigate whether the variation in abundance of specific microbial genera previously associated with sociability or autistic traits is related to macaque social behaviour. We examine the relationship between social behaviour and gut microbiome composition in a non-captive population of rhesus macaques (*Macaca mulatta*) on the island of Cayo Santiago, off Puerto Rico. Since autistic traits are continuously distributed across the human population (Wing, 1988; Constantino and Todd, 2003; Ruzich et al., 2015), it is likely that the same is true for non-human primates and indeed, macaques in this population show variation in social motivation and behaviour (Watson and Platt, 2012; Madlon-Kay et al., 2017). In particular, we use social network analysis to assess variation in social behaviour, measured through grooming interactions. Rhesus macaques live in large mixed-sex groups and the social structure of this study population is well characterised (Maestripieri and Hoffman, 2012). They are highly social animals and grooming is their primary means of making and maintaining relationships, providing a good indicator of social interactions between individuals. Our primary aim was therefore to investigate, by conducting targeted regression analyses, whether inter-individual variation in macaque social behaviour is associated with differential abundance of gut bacteria previously linked to autistic and sociability traits in rodent studies and human populations (Supplementary Table S1). We also consider the relationship between social interactions and gut microbiome similarity and diversity in this macaque population.

## Materials and methods

### Study population and behavioural data collection

Study subjects were adult rhesus macaques from a population on the 37-acre island of Cayo Santiago, off the coast of Puerto Rico (18°09′N, 65°44′W). This free-ranging population stems from a founding population introduced to the island from India in 1938 (Rawlings and Kessler, 1986). Although the animals forage for natural vegetation, their diet is also supplemented with daily feed and water but there is no regular medical intervention. Their gut microbiota has been found to be similar to captive rhesus macaques fed the same chow, indicating that the external environment has limited effect on the microbiota of these free-ranging macaques (Kuthyar et al., 2022). The macaques are individually recognised by observers who record their behaviours (primarily grooming, aggression, feeding, and resting) using 10 min focal animal observations (Altmann, 1974). Behavioural data collection is ongoing in this population and is distributed evenly during the day, both between and within subjects, with data revealing that individual social behaviour is consistent over time (Brent et al., 2013). Grooming interactions between individuals were recorded in terms of both the duration of the interaction and the identities of the giver and receiver, whilst dominance rank was calculated based on the direction of submissive interactions. Behavioural data relating to grooming interactions and social rank from years 2012 to 2013 for one social group (classified in this Cayo Santiago population as group “F”) were used in this study since this is the period over which the faecal samples were collected for microbiome sequencing. The mean time each individual’s behaviour in this group was observed was 3.2 h in 2012 and 7.8 h in 2013. Sample size was limited by the number of individuals for whom gut microbiome sequence data were available, with 50 samples in total representing 38 individuals.

### Social network metrics

Social networks based on grooming interactions (Brent et al., 2017) were constructed for each year separately. These networks included all members of the group, regardless of whether microbiome data were available for that individual. Network metrics were normalised by expressing each individual’s score relative to the mean score in each year for the social group. A sociability index was calculated for each individual by taking their cumulative score of the normalised network metrics for degree (number of different grooming partners) and strength (amount of time spent both giving and receiving grooming, relative to the amount of time for which the individual was observed).

### Sample collection, processing, and sequencing

During behavioural observation of the animals, faecal samples (uncontaminated by urine, water, or other faeces) were collected opportunistically from both sexes over the two-year period. Samples were taken shortly after defecation and then stored at −20°C. DNA was extracted from the samples according to the Earth Microbiome Project standard protocols (Gilbert et al., 2014) using the PowerSoil-HTP Kit (Qiagen). The 16S rRNA gene was amplified *via* polymerase chain reaction using universal primers for the V4 hypervariable region and the resulting amplicons were sequenced on the Illumina MiSeq platform, according to a previously described protocol (Caporaso et al., 2012). Blanks were also incorporated in sequencing runs to control for contamination. Sequencing data were processed and analysed using the software Quantitative Insights Into Microbial Ecology (QIIME) version 1.9.1 (Caporaso et al., 2010). Sequences were demultiplexed and quality filtered in QIIME and then assigned to operational taxonomic units (OTUs) using a 97% sequence similarity threshold with open-reference OTU picking against the Greengenes database (DeSantis et al., 2006). This OTU picking method was used since it preserves sequence data and therefore taxonomic diversity (Navas-Molina et al., 2013).

### Multiple regression analyses of bacterial abundance

Microbiome count data contain a high proportion of zeros (Xu et al., 2015; Weiss et al., 2017) and their frequency distributions are heavily right-skewed (Xia and Sun, 2017). This overdispersion arises because there are relatively few taxa that are found in all samples as most taxa are rare (Xu et al., 2015). Count data cannot typically be normalised using transformation and so negative binomial regression was conducted with genus abundance as the response variable (or zero-inflated negative binomial regression when the count data contained an excess of zeros). When modelling microbiome data, it is also important to take into account variation in sequencing read depth between samples. Rarefying count data or expressing the data as a proportion can result in a high rate of false positives and so is not recommended for models of taxon abundance (McMurdie and Holmes, 2014). Instead, the total read number was included as a covariate in the regression models, thereby preserving all sequence data (Goodrich et al., 2014; Xu et al., 2015).

Negative binomial regression was conducted using the R package glmmADMB to model count data. All statistical analyses in this study were performed using R 3.2.3 software (R Development Core Team, 2015). Regression models were constructed to predict genus abundance as 16S rRNA sequencing is estimated to be 96% accurate for genus identification (Srinivasan et al., 2015), whereas its phylogenetic resolution at the species level is more limited (Janda and Abbott, 2007). The main variable of interest, the sociability index, was included in each model, as well as the total read number. Individual identity was added as a random effect to control for repeated sampling of some individuals. Effects of rank, sex, age, season, and sampling year were assessed as potential confounding variables since these factors may vary with both primate microbiome composition (Degnan et al., 2012; Fogel, 2015; Bennett et al., 2016; Ren et al., 2016; Sun et al., 2016; Perofsky et al., 2017; Raulo et al., 2017; Springer et al., 2017; Hicks et al., 2018; Trosvik et al., 2018; Rudolph et al., 2022) and sociability (Schino, 2001; Krause and Ruxton, 2002; Shimizu et al., 2012; Almeling et al., 2016; Perofsky et al., 2017), and their inclusion in the regression models was guided by the Akaike information criterion (Akaike, 1974). Although rank can influence grooming relationships (Schino, 2001), it was not correlated with the sociability index in this macaque social group and so could be included in the models without resulting in multicollinearity.

### Analyses of microbiome diversity and community composition

Alpha and beta diversity analyses were conducted to investigate how microbiome diversity within and between macaques was related to grooming interactions, whilst controlling for the effects of rank, sex, age, season and sampling year. Whilst alpha diversity considers the ecological diversity of each sample individually, beta diversity measures differences in community composition between microbiome samples (Finotello et al., 2016). For these diversity analyses, microbiome count data were rarefied by randomly sampling counts without replacement so that the number of total counts for each sample was equal. This is an advisable normalisation method as uneven sequence counts across samples can significantly affect diversity estimates (Goodrich et al., 2014; Weiss et al., 2017).

A linear mixed model was constructed using the lme function in the R package nlme to predict alpha diversity, estimated with the Chao1 index based on genus-level OTUs. For these diversity analyses, we distinguished between grooming versus being groomed to determine whether microbial transmission is facilitated merely by increased contact or whether it is more the performance of grooming that facilitates transmission due to hand-to-mouth actions, similar to previous methodology studying parasite transmission (Shimizu et al., 2012). The variables rank, sex, age, season, and year were also incorporated in the model, and individual identity was included as a random effect to account for pseudoreplication.

To assess beta diversity, PERMANOVA was performed to determine which variables significantly affect the variation in microbiome composition between macaques. Gut microbial similarity between individuals based on genus-level OTUs was calculated in QIIME using both weighted UniFrac and unweighted UniFrac distance matrices (Lozupone and Knight, 2005) and visualised using EMPeror (Vázquez-Baeza et al., 2013). UniFrac distances account for the phylogenetic relatedness of microbial taxa; unweighted distances only consider presence versus absence of taxa, whereas weighted distances also take into account their abundance. PERMANOVA was carried out on these matrices using the adonis function from the R package vegan with 1,000 permutations, incorporating the same variables as the model predicting alpha diversity. This function estimates the variance in the distance matrix attributable to each variable.

## Results

### Taxonomic composition of the microbiome

Gut microbiome composition differed considerably between individuals (Supplementary Figure S1), as has been found in numerous other studies of primate social groups (Fogel, 2015; Tung et al., 2015; Ren et al., 2016; Perofsky et al., 2017). The most abundant genera across all samples were similar to previous research on the gut microbiome of captive rhesus macaques (Yasuda et al., 2015; Amaral et al., 2017).

### Regression models of bacterial abundance

**Figure 1:**
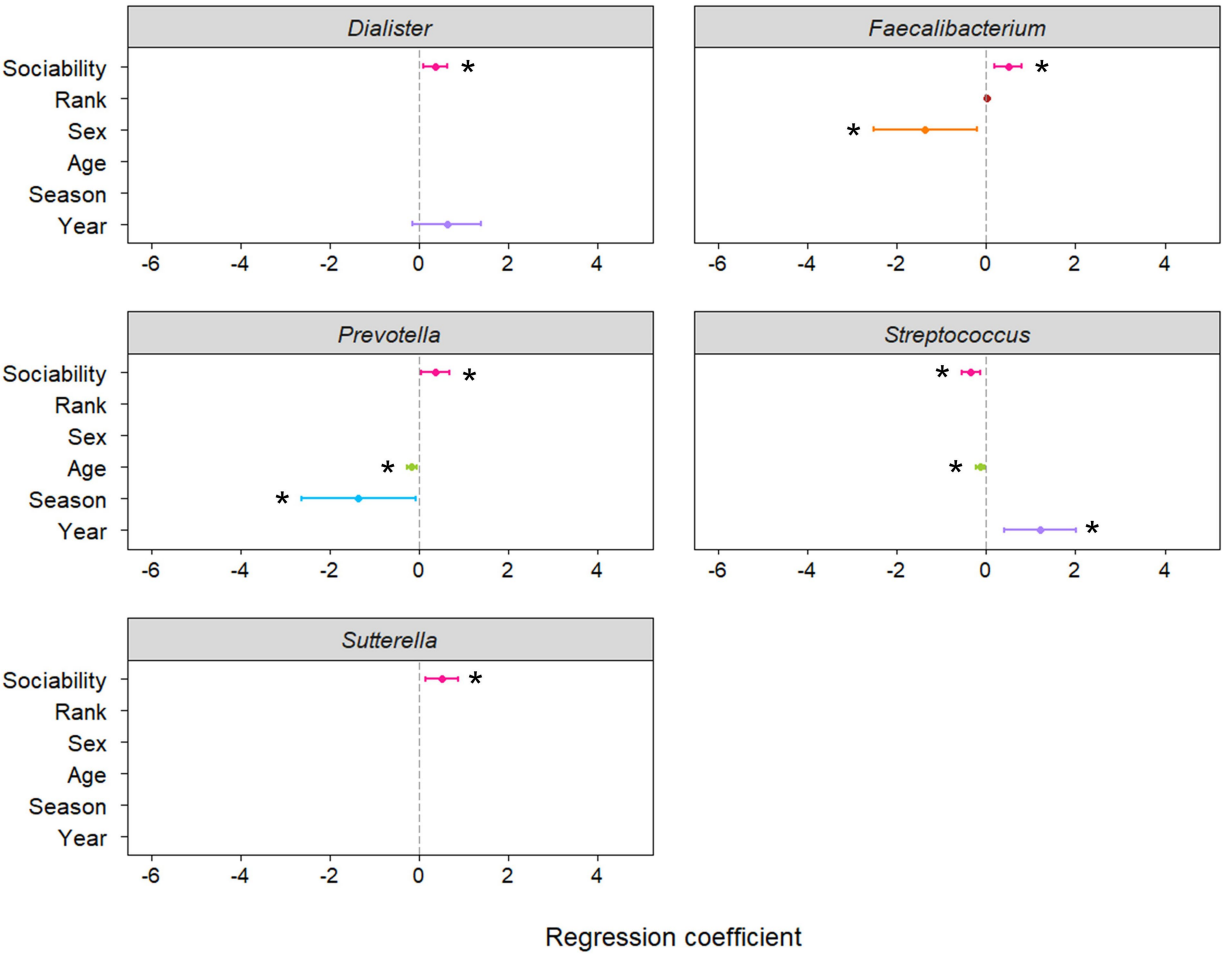
Coefficient plots from regression models predicting abundance of genera in the macaque gut microbiome. Asterisks denote significant predictors of genus abundance at α = 0.05 and bars indicate 95% confidence intervals. The main variable of interest was the sociability index (as measured by the number of grooming partners and duration of grooming interactions), whilst other variables were controlled for where appropriate, as determined using Akaike information criterion. A positive coefficient for sex indicates a higher abundance of the genus in females and a positive coefficient for season indicates a higher abundance of the genus during the mating season than the birthing season. Plots displayed here depict genera whose abundance was significantly related to sociability (for remaining regression coefficient plots see Supplementary Figure S2).

**Figure 2:**
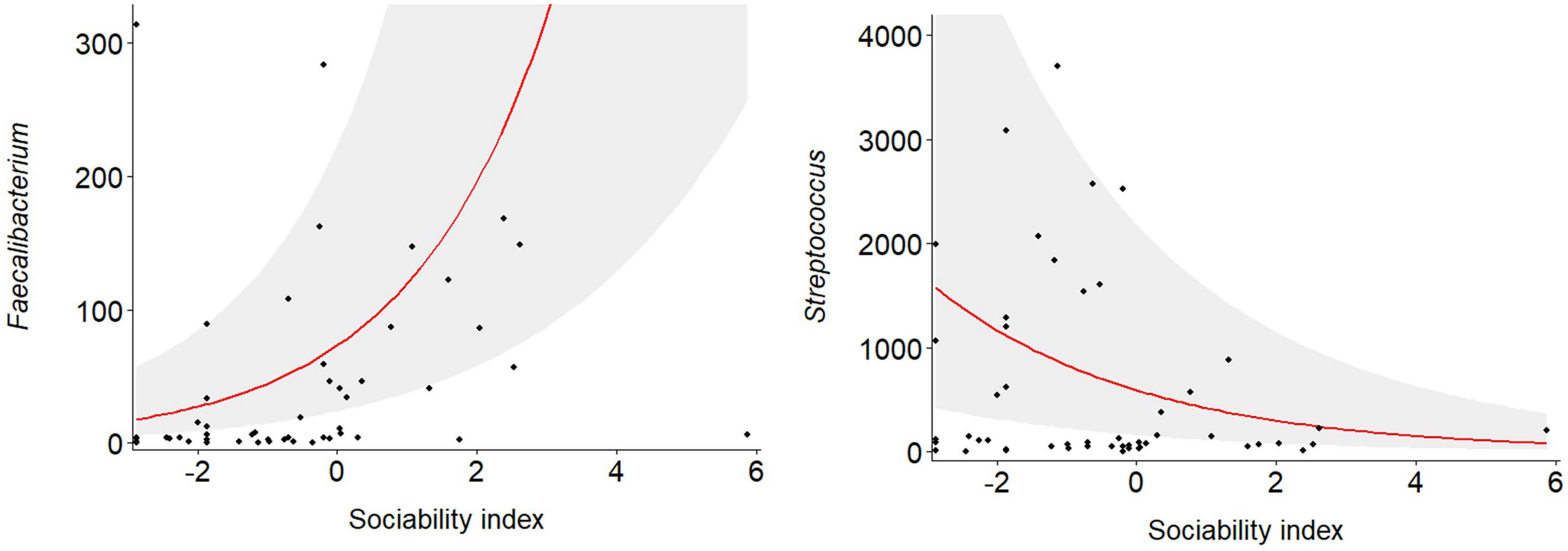
Relationship between macaque sociability and bacterial abundance for the two most strongly associated genera, *Faecalibacterium* and *Streptococcus*, as predicted from negative binomial regression. Shaded region denotes 95% confidence intervals for the regression line.

Negative binomial regression analyses revealed that the abundance of five of the 16 genera were significantly predicted by host sociability (Figure 1; Supplementary Table S2). Sociability positively predicted genus abundance for *Dialister* (*p* = 0.008), *Faecalibacterium* (*p* = 0.002), *Prevotella* (*p* = 0.019), and *Sutterella* (*p* = 0.006). On the other hand, the abundance of *Streptococcus* was negatively predicted by sociability (*p* = 0.001). Since we conducted targeted regression analyses based on specific genera identified in the literature, rather than an exploratory study of all genera constituting the macaque gut microbiome, the value of applying corrections for multiple comparisons is questionable. However, if we do apply this more conservative threshold to our results using the Benjamini–Hochberg method then the significant relationship between sociability and bacterial abundance is maintained for the strongest associations, namely with *Faecalibacterium* and *Streptococcus* (Figure 2).

In terms of the other variables controlled for in the models, sex, age, season, and sampling year were also significantly related to the abundance of certain genera (Supplementary Table S2). Notably, *Faecalibacterium* was less abundant in females compared to males and age was negatively related to the abundance of *Prevotella* and *Streptococcus*.

### Effects of grooming interactions on microbiome diversity and community composition

**Figure 3:**
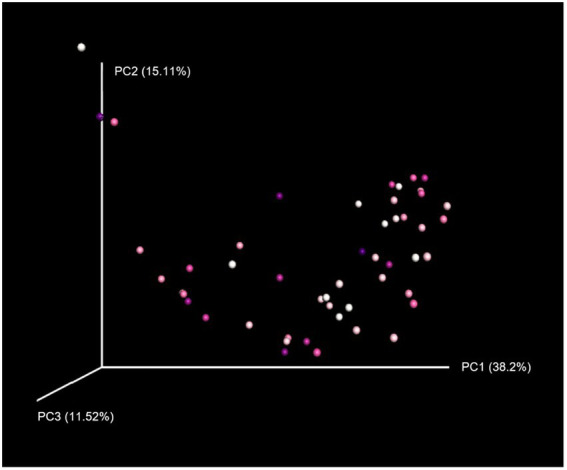
Principal coordinates analysis plot of microbiome composition using weighted UniFrac distances. Principal coordinates analysis reduces the dimensionality of microbiome data so that differences in microbiome composition between samples can be visualised, with points clustered together indicating more similar gut microbial communities. Points are coloured on a gradient from white (individuals receiving the least grooming) to dark purple (those receiving the most grooming).

Neither grooming interactions nor any of the other variables included in the linear mixed model significantly predicted alpha diversity of the gut microbiome (Supplementary Table S3). In terms of beta diversity, the amount of grooming received by an individual explained 5% of the variation in gut microbiome composition between individuals (*p* = 0.022), as revealed by PERMANOVA using weighted UniFrac distances (Figure 3). Age (*p* = 0.039) and season (*p* = 0.007) were also significant factors influencing microbiome composition. However, for unweighted UniFrac distances (Supplementary Table S4) only season had a significant effect (*p* = 0.015).

## Discussion

We show that a number of genera previously linked to individual differences in sociability or autistic traits are also significantly related to macaque social behaviour in this population. The majority of significant relationships were in the expected direction based on the literature (Supplementary Table S1). The genera *Dialister*, *Faecalibacterium*, *Prevotella*, and *Sutterella* were more abundant in individuals scoring higher on the sociability index, that is, those engaging in grooming interactions for a longer duration and with more partners.

In this macaque population, *Streptococcus* was found to be more abundant in less sociable individuals. Notably, some members of this genus can be opportunistic pathogens (Henriques-Normark and Normark, 2010), producing pro-inflammatory cytokines (van den Bogert et al., 2014), and thus increased *Streptococcus* abundance in less sociable animals may be related to poorer social integration, greater social stress, and poorer health. In fact, elevated glucocorticoids (indicating a heightened stress response) were recently found to be associated with an increased abundance of members of the family Streptococcaceae in wild North American red squirrels (Petrullo et al., 2022). There is also evidence that this genus is more prevalent in humans suffering from depression (Lin et al., 2017). A higher abundance of *Streptococcus* in less sociable macaques may therefore reflect the presence of potentially pathogenic members of this genus.

Notable findings in terms of the other variables controlled for in the models include a lower abundance of *Faecalibacterium* in females compared to males, in accordance with findings in humans (de Cárcer et al., 2011). Older animals showed reduced abundance of *Prevotella* and *Streptococcus*. Similarly, in humans, *Prevotella* has been found to be more prevalent in infants than adults (Yatsunenko et al., 2012) and its abundance is negatively predicted by age (Johnson, 2020).

It is compelling that some of the gut bacterial genera that are generally reduced in abundance in autism also have a lower abundance in less sociable monkeys. Whilst this may in part reflect an effect of gut microorganisms on the brain and behaviour, as has been repeatedly demonstrated in animal models (Cryan and Dinan, 2012; Sampson and Mazmanian, 2015; Sharon et al., 2016), it is important to consider the reciprocal interactions that may be involved. The relationships reported here between macaque social behaviour and bacterial abundance may reflect the fact that certain microorganisms are better adapted for social transmission and thus tend to have a greater abundance in more sociable individuals. For example, the genus *Prevotella* was previously found to be positively associated with host density in a wild animal population (Li et al., 2016), suggesting that it may be transmitted effectively through social contact. In addition to the social transmission of microorganisms, another factor that can influence the gut microbiome is exposure to stress (Bailey and Coe, 1999; Bailey et al., 2011; Bangsgaard Bendtsen et al., 2012; Bharwani et al., 2016). Grooming interactions have been shown to reduce heart rate and cortisol levels, thereby buffering the detrimental effects of stress (Brent et al., 2014). This may therefore represent another route through which social behaviour may be linked to gut microbiome composition.

Regardless of the direction of these relationships, it is notable that several of these bacterial genera related to sociability, such as *Faecalibacterium* and *Prevotella*, have also been associated with beneficial effects on host immunity. The positive relationship between sociability and the abundance of *Faecalibacterium* is particularly interesting since this genus is well known for its potent anti-inflammatory properties (Qiu et al., 2013; Quévrain et al., 2015) and is often associated with good health (Miquel et al., 2013). Indeed, its abundance in humans is typically reduced in illnesses such as inflammatory bowel disease (Sokol et al., 2008; Martín et al., 2015), chronic fatigue syndrome (Giloteaux et al., 2016), long covid (Liu et al., 2022), depression (Jiang et al., 2015), and bipolar disorder (Evans et al., 2017). This genus is only known to contain one species, *F*. *prausnitzii*, which is one of the most abundant bacterial species in the human gut microbiome (Walker et al., 2011; Lopez-Siles et al., 2012; Johnson, 2020). It is a prolific producer of the short-chain fatty acid butyrate (Louis and Flint, 2009), which has beneficial effects on the immune system and there is some evidence from animal studies that it can cross the blood–brain barrier and thereby influence brain development and behaviour (Rogers et al., 2016; Stilling et al., 2016). In addition, a higher abundance of *Prevotella* in young macaques has been associated with an increased number of regulatory T cells, which are important for host defence against pathogens (Ardeshir et al., 2014). Indeed, higher levels of *Prevotella* are correlated with a less permeable gut barrier (Fields et al., 2018). *Prevotella* can produce the anti-inflammatory short-chain fatty acid propionate (Cosorich et al., 2017) and this genus has been shown to have beneficial anti-inflammatory effects in animal models of disease (Marietta et al., 2016; Mangalam et al., 2017). In fact, many of the genera targeted in these regression models due to their associations with sociability or autistic traits are important producers of short-chain fatty acids that can affect host physiology, including the brain (Koh et al., 2016).

Our finding that engagement in social interactions is positively related to the abundance of gut microorganisms with beneficial immunological functions (and negatively related to potentially pathogenic members of the microbiota) is pertinent to the well-known association between social relationships and health, which is postulated to be mediated at least in part by the immune system (Holt-Lunstad et al., 2010; Yang et al., 2016). In fact, research which manipulated the social status of female macaques found that a lower rank and reduced engagement in grooming interactions resulted in a heightened inflammatory response (Snyder-Mackler et al., 2016). In addition, recent research on Cayo Santiago macaques has found that individuals who are more socially connected, with a greater number of grooming partners, tend to have fewer white blood cells, indicating lower levels of inflammation (Pavez-Fox et al., 2021). Given the important anti-inflammatory effects of certain members of the microbiota such as *Faecalibacterium*, the gut microbial community may play a role in mediating the relationship between social integration and health *via* its regulation of the host immune response. It would be informative therefore for future research to assess inflammatory markers in conjunction with the microbiome to test whether host immune status is related to the abundance of these bacterial genera that are associated with host social behaviour. Indeed, examining the potential links between the microbiome, health and sociality has been identified as an important research direction (Nunn et al., 2015) and the findings here provide some insight into these interrelations.

In terms of the diversity analyses, none of the variables significantly predicted alpha diversity of the gut microbiome. This contrasts some previous findings that gut microbiome diversity is positively related to sociability in primate social groups (Li et al., 2016; Moeller et al., 2016; Billiet et al., 2017; Perofsky et al., 2017) and also to social network size in humans (Johnson, 2020). Perhaps this result reflects the limited sample size, although other primate studies have likewise reported no relationship between sociability and diversity (Bennett et al., 2016), or even a negative relationship (Raulo et al., 2017). Previous research in primates has also found no effect of age or sex on gut microbiome composition (Raulo et al., 2017; Rudolph et al., 2022) or diversity (Bridgewater et al., 2017), whilst studies that have found an effect typically report small effect sizes (Amato et al., 2014; Tung et al., 2015; Bennett et al., 2016). Although microbiome diversity has been linked to seasonal differences in primates (Sun et al., 2016; Raulo et al., 2017; Springer et al., 2017; Trosvik et al., 2018; Rudolph et al., 2022) and Hadza hunter-gatherers (Schnorr et al., 2014), there was no evidence that season significantly influenced diversity in this macaque population.

In contrast to alpha diversity, social relationships were found to predict beta diversity of the gut microbiome in this population. Specifically, the amount of grooming an individual received was a significant factor explaining differences in microbiome composition between macaques. This is consistent with previous research showing that social interactions such as grooming can promote gut microbiome similarity within social groups (Tung et al., 2015; Moeller et al., 2016; Raulo et al., 2017). Rhesus macaques are not coprophagic and so grooming is likely the primary method of microbial transmission between individuals through hand-to-mouth actions. The results revealed that the amount of grooming an individual received can explain approximately 5% of the variation in gut microbiome composition between individuals, which is not negligible given the many different factors that can influence the microbiome. However, this result was only significant using weighted, and not unweighted, UniFrac distances. Previous research has also shown that beta diversity analyses on weighted versus unweighted UniFrac distances can yield significantly different results (Lozupone et al., 2007). In particular, weighted UniFrac is effective at detecting differences in community composition that are due to differential microbial abundance, rather than merely the presence or absence of certain taxa as measured with unweighted UniFrac (Navas-Molina et al., 2013). This therefore suggests that grooming may be more related to the abundance of particular microbial taxa, rather than which taxa are present in the gut. We also found evidence that season and age can significantly influence beta diversity of the microbial community, in accordance with previous research (Sun et al., 2016; Springer et al., 2017; Hicks et al., 2018; Trosvik et al., 2018). Notably, there was no effect of dominance rank on alpha or beta diversity of the microbiome. This is somewhat surprising as social status is known to impact primate health and immunity (Sapolsky, 2005; Archie et al., 2012; Tung and Barreiro, 2012; Snyder-Mackler et al., 2016) and so may also be expected to influence the host’s microbiome. This suggests that the gut microbial community may be more strongly associated with an individual’s engagement in social interactions than social rank.

The reciprocal interaction between host behaviour and the gut microbiome presents a challenging case to disentangle cause from effect. Whilst experiments with laboratory animals are useful for manipulating the gut microbiome to examine the causal relationships involved, the very hygienic nature of laboratory environments has implications for host physiology, most notably a weakened immune system (Beura et al., 2016; Abolins et al., 2017; Rosshart et al., 2017). Thus, one benefit of research in non-captive animal populations is that it represents a more ecologically valid system for understanding how behaviour might be influenced by the microbiome and vice versa (Allen et al., 2017). Given the demonstrated effects of the gut microbiome on rodent behaviour, it is certainly plausible, if not expected, that the gut microbiome may influence behaviour of other mammalian species, with implications for behavioural ecology and evolution (Amato, 2016). However, further research is required to determine the causal relationships between the gut microbiome and social behaviour in non-captive animal populations. In fact, since probiotic supplementation has been shown to increase sociability in rodent models (Buffington et al., 2016; Bharwani et al., 2017), it would be interesting to test whether probiotics increase social motivation and engagement in grooming interactions in the macaque population. This semi-wild population provides the rare opportunity for conducting intervention studies in a natural environment.

## Conclusion

This work adds to the growing research at the interface of microbial ecology and animal behaviour but also extends it in a novel direction. This is the first study to investigate whether microbial taxa previously associated with sociability or autistic traits are differentially abundant in an animal population with respect to individual variation in social behaviour. Our findings advance our understanding of how gut microbial communities may be associated with host behaviour and reveal that social relationships may be intertwined with health and immune status *via* the microbiota.

## Data availability statement

The original contributions presented in the study are included in the article/Supplementary material; further inquiries can be directed to the corresponding author. The sequencing data are deposited in the EBI repository, accession number ERP141557.

## Ethics statement

All applicable ethical guidelines for the care and use of animals were followed. All the data analysed in this study had previously been collected according to research procedures approved by the Caribbean Primate Research Centre and the Institutional Animal Care and Use Committee (IACUC) of the University of Puerto Rico.

## Author contributions

KJ conceived the idea, analysed, interpreted, and presented the data and wrote the manuscript. KW processed the samples and provided the raw microbiome data. KJ and KW discussed the methodology, results, and the draft manuscript. RD and PB supervised the project. All authors contributed to the article and approved the submitted version.

## Funding

KJ was funded by BBSRC (BB/J014427/1). Data provided from the macaque population were collected under funding from the NIH (R01MH096875 and R01MH089484). The Caribbean Primate Research Centre is supported by the NIH and an Animal and Biological Material Resource Centre Grant (P40OD012217) awarded to the University of Puerto Rico from the Office of Research Infrastructure Programmes (ORIP).

## Conflict of interest

The authors declare that the research was conducted in the absence of any commercial or financial relationships that could be construed as a potential conflict of interest.

## Publisher’s note

All claims expressed in this article are solely those of the authors and do not necessarily represent those of their affiliated organizations, or those of the publisher, the editors and the reviewers. Any product that may be evaluated in this article, or claim that may be made by its manufacturer, is not guaranteed or endorsed by the publisher.
