# Supplementary material for: Sociability in a non-captive macaque population is associated with beneficial gut bacteria
Source: Front Microbiol. 2022 Nov 11;13:1032495. doi: 10.3389/fmicb.2022.1032495 (PMC9691693; doi:10.3389/fmicb.2022.1032495)
Supplement: Supplementary file 1 [file Data_Sheet_1.pdf]

Sociability in a non-captive macaque population is associated with beneficial gut bacteria

Katerina V.-A. Johnson, Karli K. Watson, Robin I. M. Dunbar and Philip W. J. Burnet

Supplementary Material

1 Supplementary data

See Supplementary data files uploaded.

2 Supplementary figures

See next page.

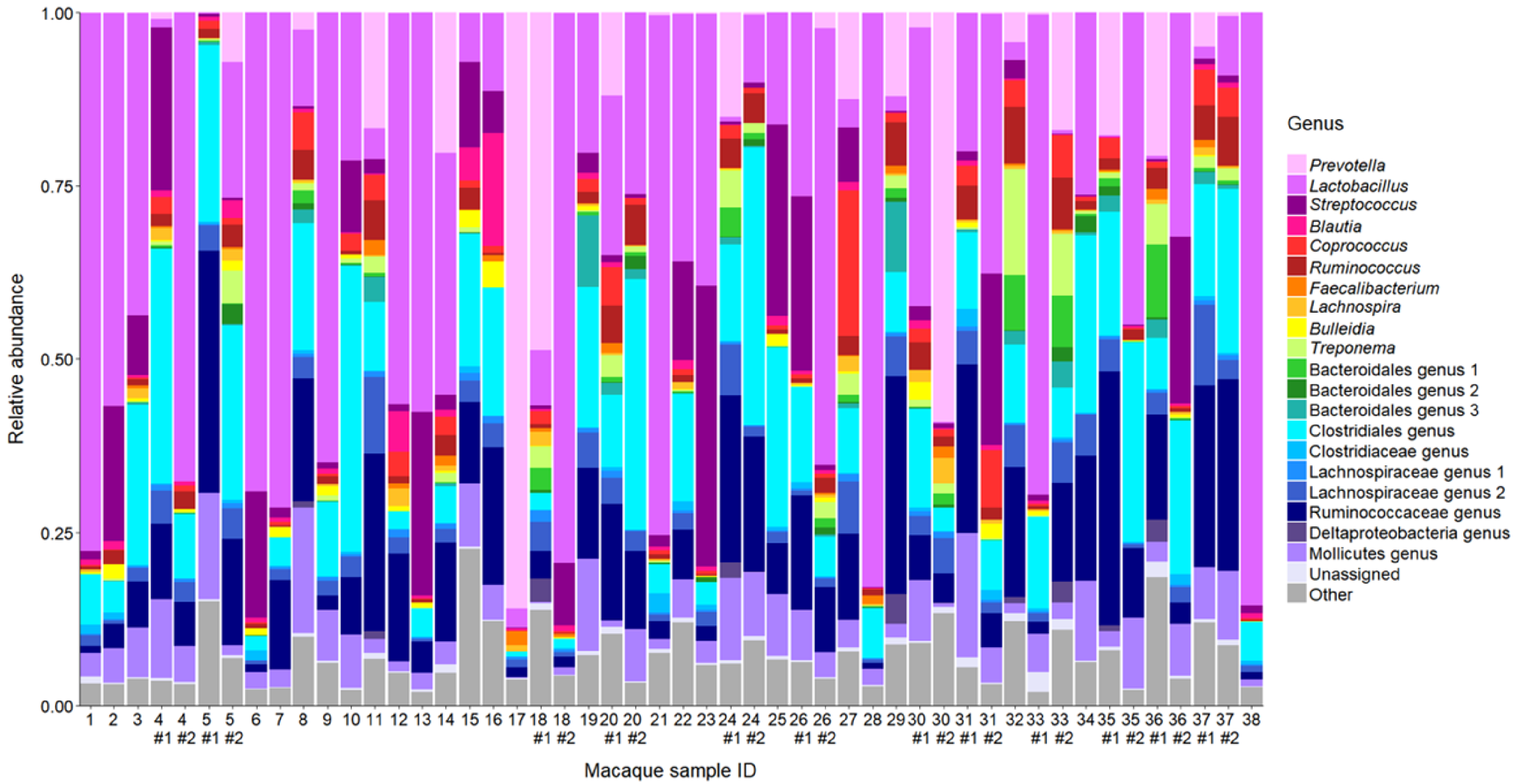

Figure S1. Stacked bar plot summarizing gut microbiome composition at genus level for the macaque study population. Microbiome data are plotted showing relative abundance of the top twenty most abundant genera across all samples, with the remaining genera grouped into 'Other'. The twenty genera in the key are ordered from most to least abundant. As labelled in the chart, some individuals from the population were sampled twice on different occasions (such as a different year or season). Microbial taxa that could not be identified to genus level were instead numbered at the taxonomic rank to which they could be resolved. By convention, 'Unassigned' denotes reads not sufficiently similar to any previously classified microbial taxa in the Greengenes database.

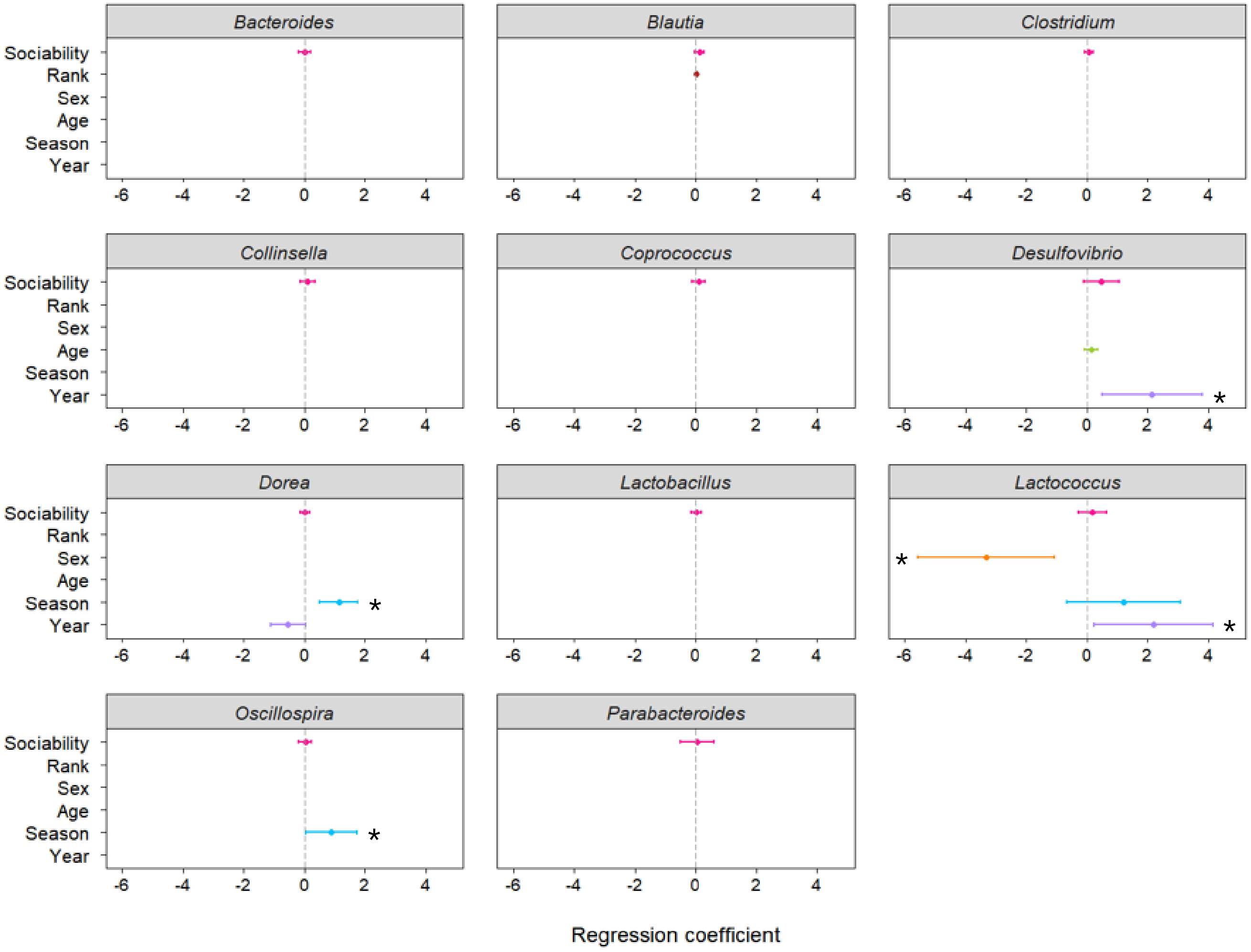

Figure S2. Coefficient plots from regression models predicting abundance of genera in the gut microbiome. Asterisks denote significant predictors of genus abundance at $\alpha = 0.05$ and bars indicate 95% confidence intervals. The main variable of interest was the sociability index (as measured by the number of grooming partners and duration of grooming interactions), while other variables were also controlled for where appropriate, as determined using Akaike information criterion. A positive coefficient for sex indicates a higher abundance of the genus in females and a positive coefficient for season indicates a higher abundance of the genus during the mating season than the birthing season. Plots displayed here depict genera whose abundance was not significantly related to sociability (for significant results see Figure 1).

3 Supplementary tables

Table S1. Summary of significant associations between bacterial genera endogenous in the gut and sociability or autistic traits reported in the literature. Table lists findings from research in both animal models and human populations and includes all genera associated with autism or social behaviour in at least two independent studies (adapted from Johnson, 2020). Arrows depict whether an increased or decreased abundance has been associated with either sociability or autistic traits.

Genus	Change in abundance	Sociability/ autistic traits	Study subject	References
Bacteroides	↑	Autism	Children	de Angelis *et al*., 2013; Finegold *et al*., 2010
Blautia	↓	Autism	Infants	Inoue *et al*., 2016
	↓	Autism	Children	Luna *et al*., 2017
	↓	Autism	Mice	Golubeva *et al*., 2017
Clostridium	↑	Autism	Children	de Angelis *et al*., 2013; Finegold *et al*., 2002; Grimaldi *et al*., 2017; Luna *et al*., 2017; Parracho *et al*., 2005; Song *et al*., 2004; Strati *et al*., 2017; Tomova *et al*., 2015
	↓	Autism	Children	Finegold *et al*., 2010
Collinsella	↑	Autism	Children	Strati *et al*., 2017
	↓	Autism	Children	Finegold *et al*., 2010
Coprococcus	↓	Autism	Children	de Angelis *et al*., 2013; Kang *et al*., 2013
Desulfovibrio	↑	Autism	Children	Finegold *et al*., 2010; Tomova *et al*., 2015
	↓	Sociability	Adults	Johnson, 2020
Dialister	↓	Autism	Children	Finegold *et al*., 2010; Strati *et al*., 2017
	↑	Sociability	Infants	Christian *et al*., 2015
Dorea	↑	Autism	Children	de Angelis *et al*., 2013; Strati *et al*., 2017
	↓	Autism	Children	Luna *et al*., 2017
Faecalibacterium	↑	Autism	Infants	Inoue *et al*., 2016
	↓	Autism	Children	de Angelis *et al*., 2013; Kang *et al*., 2018
Lactobacillus	↑	Autism	Children	Adams *et al*., 2011; Kang *et al*., 2013; Pulikkan *et al*., 2018; Strati *et al*., 2017; Tomova *et al*., 2015
	↓	Autism	Children	de Angelis *et al*., 2013
Lactococcus	↓	Autism	Children	de Angelis *et al*., 2013; Finegold *et al*., 2010
	↑	Sociability	Adults	Johnson, 2020
Oscillospira	↓	Autism	Children	de Angelis *et al*., 2013
	↑	Sociability	Adults	Johnson, 2020
	↑	Sociability	Mice	Szyszkowicz *et al*., 2017
Parabacteroides	↓	Autism	Children	Strati *et al*., 2017
	↑	Autism	Children	Finegold *et al*., 2010
	↑	Sociability	Infants	Christian *et al*., 2015
Prevotella	↑	Autism	Children	de Angelis *et al*., 2013
	↓	Autism	Children	Kang *et al*., 2013
Streptococcus	↓	Autism	Children	de Angelis *et al*., 2013; Finegold *et al*., 2010
Sutterella	↑	Autism	Children	Wang *et al*., 2011, 2013; Williams *et al*., 2011, 2012
	↓	Autism	Children	Luna *et al*., 2017
	↓	Sociability	Adults	Johnson, 2020

See accompanying spreadsheet for Tables S2-S4:

Table S2. Output from regression models predicting abundance of genera in the gut microbiome. Highlighted cells denote significant predictors of genus abundance where $P < 0.05$. CL represents 95% confidence limit for each regression coefficient.

Table S3. Results of linear mixed model to test the factors affecting gut microbiome diversity, as estimated using the Chao1 index.

Table S4. Results of PERMANOVA to test the factors affecting gut microbial community composition. PERMANOVA with 1,000 permutations was conducted on both weighted and unweighted UniFrac distance matrices. Highlighted cells denote significant associations where $P < 0.05$.

4 Supplementary references

Adams, J. B., Johansen, L. J., Powell, L. D., Quig, D., and Rubin, R. A. (2011). Gastrointestinal flora and gastrointestinal status in children with autism – comparisons to typical children and correlation with autism severity. *BMC Gastroenterol.* 11:22. doi: 10.1186/1471-230X-11-22

de Angelis, M., Piccolo, M., Vannini, L., Siragusa, S., de Giacomo, A., Serrazzanetti, I., *et al*. (2013). Fecal microbiota and metabolome of children with autism and pervasive developmental disorder not otherwise specified. *PLoS One* 8:e76993. doi: 10.1371/journal.pone.0076993

Christian, L. M., Galley, J. D., Hade, E. M., Schoppe-Sullivan, S., Kamp Dush, C., and Bailey, M. T. (2015). Gut microbiome composition is associated with temperament during early childhood. *Brain. Behav. Immun.* 45, 118–127. doi: 10.1016/j.bbi.2014.10.018

Finegold, S. M., Dowd, S. E., Gontcharova, V., Liu, C., Henley, K. E., Wolcott, R. D., *et al*. (2010). Pyrosequencing study of fecal microflora of autistic and control children. *Anaerobe* 16, 444–453. doi: 10.1016/j.anaerobe.2010.06.008

Finegold, S. M., Molitoris, D., Song, Y., Liu, C., Vaisanen, M., Bolte, E., *et al*. (2002). Gastrointestinal microflora studies in late-onset autism. *Clin. Infect. Dis.* 35 (Suppl. 1), S6–S16. doi: 10.1086/341914.

Golubeva, A. V., Joyce, S. A., Moloney, G., Burokas, A., Sherwin, E., Arboleya, S., *et al*. (2017). Microbiota-related changes in bile acid & tryptophan metabolism are associated with gastrointestinal dysfunction in a mouse model of autism. *EBioMedicine* 24, 166–178. doi: 10.1016/j.ebiom.2017.09.020

Grimaldi, R., Cela, D., Swann, J. R., Vulevic, J., Gibson, G. R., Tzortzis, G., *et al*. (2017). *In vitro* fermentation of B-GOS: impact on faecal bacterial populations and metabolic activity in autistic and non-autistic children. *FEMS Microbiol. Ecol.* 93:fiw23. doi: 10.1093/femsec/fiw233

Inoue, R., Sakaue, Y., Sawai, C., Sawai, T., Ozeki, M., Romero-Pérez, G. A., *et al*. (2016). A preliminary investigation on the relationship between gut microbiota and gene expressions in peripheral mononuclear cells of infants with autism spectrum disorders. *Biosci. Biotechnol. Biochem.* 80, 2450–2458. doi: 10.1080/09168451.2016.1222267

Johnson, K. V.-A. (2020). Gut microbiome composition and diversity are related to human personality traits. *Hum. Microbiome J.* 15:100069. doi: 10.1016/j.humic.2019.100069

Kang, D.-W., Ilhan, Z. E., Isern, N. G., Hoyt, D. W., Howsmon, D. P., Shaffer, M., *et al*. (2018). Differences in fecal microbial metabolites and microbiota of children with autism spectrum disorders. *Anaerobe* 49, 121–131. doi: 10.1016/j.anaerobe.2017.12.007

Kang, D., Park, J. G., Ilhan, Z. E., Wallstrom, G., Labaer, J., Adams, J. B., *et al*. (2013). Reduced incidence of *Prevotella* and other fermenters in intestinal microflora of autistic children. *PLoS One* 8:e68322. doi: 10.1371/journal.pone.0068322

Luna, R. A., Oezguen, N., Balderas, M., Venkatachalam, A., Runge, J. K., Versalovic, J., *et al*. (2017). Distinct microbiome–neuroimmune signatures correlate with functional abdominal pain in children with autism spectrum disorder. *Cell. Mol. Gastroenterol. Hepatol.* 3, 218–230. doi: 10.1016/j.jcmgh.2016.11.008

Parracho, H. M. R. T., Bingham, M. O., Gibson, G. R., and McCartney, A. L. (2005). Differences between the gut microflora of children with autistic spectrum disorders and that of healthy children. *J. Med. Microbiol.* 54, 987–991. doi: 10.1099/jmm.0.46101-0

Pulikkan, J., Maji, A., Dhakan, D. B., Saxena, R., Mohan, B., Anto, M. M., *et al*. (2018). Gut microbial dysbiosis in Indian children with autism spectrum disorders. *Microb. Ecol.* 76, 1102–1114. doi: 10.1007/s00248-018-1176-2

Song, Y., Liu, C., and Finegold, S. M. (2004). Real-time PCR quantitation of Clostridia in feces of autistic children. *Appl. Environ. Microbiol.* 70, 6459–6465. doi: 10.1128/AEM.70.11.6459

Strati, F., Cavalieri, D., Albanese, D., Felice, C. De, Donati, C., Hayek, J., *et al*. (2017). New evidences on the altered gut microbiota in autism spectrum disorders. *Microbiome* 5:24. doi: 10.1186/s40168-017-0242-1

Szyszkowicz, J. K., Wong, A., Anisman, H., Merali, Z., and Audet, M.-C. (2017). Implications of the gut microbiota in vulnerability to the social avoidance effects of chronic social defeat in male mice. *Brain. Behav. Immun.* 66, 45–55. doi: 10.1016/j.bbi.2017.06.009

Tomova, A., Husarova, V., Lakatosova, S., Bakos, J., Vlkova, B., Babinska, K., *et al*. (2015). Gastrointestinal microbiota in children with autism in Slovakia. *Physiol. Behav.* 138, 179–187. doi: 10.1016/j.physbeh.2014.10.033

Wang, L., Christophersen, C. T., Sorich, M. J., Gerber, J. P., Angley, M. T., and Conlon, M. A. (2011). Low relative abundances of the mucolytic bacterium *Akkermansia muciniphila* and *Bifidobacterium* spp. in feces of children with autism. *Appl. Environ. Microbiol.* 77, 6718–6721. doi: 10.1128/AEM.05212-11

Wang, L., Christophersen, C. T., Sorich, M. J., Gerber, J. P., Angley, M. T., and Conlon, M. A. (2013). Increased abundance of *Sutterella* spp. and *Ruminococcus* torques in feces of children with autism spectrum disorder. *Mol. Autism* 4:42. doi: 10.1186/2040-2392-4-42

Williams, B. L., Hornig, M., Buie, T., Bauman, M. L., Paik, M. C., Bennett, A., *et al*. (2011). Impaired carbohydrate digestion and transport and mucosal dysbiosis in the intestines of children with autism and gastrointestinal disturbances. *PLoS One* 6:e24585. doi: 10.1371/journal.pone.0024585

Williams, B. L., Hornig, M., Parekh, T., and Lipkin, W. I. (2012). Application of novel PCR-based methods for detection, quantitation, and phylogenetic characterization of *Sutterella* species in intestinal biopsy samples from children with autism and gastrointestinal disturbances. *MBio* 3:e00261-11. doi: 10.1128/mBio.00261-11
